# Chemotherapy‐Enabled Colorectal Cancer Immunotherapy of Self‐Delivery Nano‐PROTACs by Inhibiting Tumor Glycolysis and Avoiding Adaptive Immune Resistance

**DOI:** 10.1002/advs.202309204

**Published:** 2024-01-18

**Authors:** Lin‐Ping Zhao, Rong‐Rong Zheng, Xiao‐Na Rao, Chu‐Yu Huang, Hang‐Yu Zhou, Xi‐Yong Yu, Xue‐Yan Jiang, Shi‐Ying Li

**Affiliations:** ^1^ Key Laboratory of Biological Targeting Diagnosis Therapy and Rehabilitation of Guangdong Higher Education Institutes The Fifth Affiliated Hospital Guangzhou Medical University Guangzhou 510700 P. R. China; ^2^ The Fifth Affiliated Hospital Guangdong Provincial Key Laboratory of Molecular Target & Clinical Pharmacology the NMPA and State Key Laboratory of Respiratory Disease the School of Pharmaceutical Sciences Guangzhou Medical University Guangzhou 511436 P. R. China; ^3^ Department of Pulmonary and Critical Care Medicine Zhujiang Hospital Southern Medical University Guangzhou 510280 P. R. China

**Keywords:** chemotherapy, colorectal cancer, immunotherapy, nano‐PROTACs, self‐delivery

## Abstract

The chemo‐regulation abilities of chemotherapeutic medications are appealing to address the low immunogenicity, immunosuppressive lactate microenvironment, and adaptive immune resistance of colorectal cancer. In this work, the proteolysis targeting chimera (PROTAC) of BRD4 (dBET57) is found to downregulate colorectal cancer glycolysis through the transcription inhibition of c‐Myc, which also inhibits the expression of programmed death ligand 1 (PD‐L1) to reverse immune evasion and avoid adaptive immune resistance. Based on this, self‐delivery nano‐PROTACs (designated as DdLD NPs) are further fabricated by the self‐assembly of doxorubicin (DOX) and dBET57 with the assistance of DSPE‐PEG_2000_. DdLD NPs can improve the stability, intracellular delivery, and tumor targeting accumulation of DOX and dBET57. Meanwhile, the chemotherapeutic effect of DdLD NPs can efficiently destroy colorectal cancer cells to trigger a robust immunogenic cell death (ICD). More importantly, the chemo‐regulation effects of DdLD NPs can inhibit colorectal cancer glycolysis to reduce the lactate production, and downregulate the PD‐L1 expression through BRD4 degradation. Taking advantages of the chemotherapy and chemo‐regulation ability, DdLD NPs systemically activated the antitumor immunity to suppress the primary and metastatic colorectal cancer progression without inducing any systemic side effects. Such self‐delivery nano‐PROTACs may provide a new insight for chemotherapy‐enabled tumor immunotherapy.

## Graphical abstract

Self‐delivery nano‐PROTACs (designated as DdLD NPs) are fabricated by the self‐assembly of doxorubicin and dBET57 with the assistance of DSPE‐PEG_2000_. Chemo‐regulation effect of DdLD NPs can inhibit glycolysis to improve immunosuppressive lactate microenvironment, and simultaneously downregulate PD‐L1 expression to decrease adaptive immune resistance, thereby systemically activating the immunotherapy of metastatic colorectal cancer.

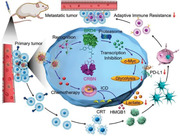

## 1 Introduction

Colorectal cancer is one of the most commonly diagnosed cancer with high mortality. Effective medication strategies with long‐lasting benefits are desired in consideration of its characteristics of easy metastasis.^[^ ^1^ ^]^ As we know, the upregulation of programmed death ligand 1 (PD‐L1) enables colorectal cancer cells to escape from immune elimination, but immune checkpoint blockade (ICB) can reverse the immune inhibitory signals to provoke durable antitumor immunity.^[^ ^2^ ^]^ Beneficially, ICB can reinvigorate cytotoxic T lymphocytes (CTLs) to secrete antitumor cytokines (perforin, granzyme, interferon γ, et al.), establishing an immunological memory and protective antitumor immunity for metastatic tumor eradication.^[^ ^3^ ^]^ However, the immune checkpoint inhibitors of clinically available antibodies exhibit poor bioavailability, which have a limited response rate in colorectal cancer due to the low immunogenicity and immunosuppressive tumor microenvironment (ITM).^[^ ^4^ ^]^ Of note, the glucose metabolic derangement is the most indicated feature to cause lactate accumulation, which will directly suppress immune cell function and indirectly recruit immunosuppressive cell types to impair antitumor immunity.^[^ ^5^ ^]^ Moreover, CTLs produced interferon γ (IFN‐γ) can feedback upregulate PD‐L1 on colorectal cancer cell surface, further exacerbating the immune evasion and inducing adaptive immune resistance during tumor immunotherapy.^[^ ^6^ ^]^ Therefore, a therapeutic synergism is urgently needed to inhibit tumor glycolysis and avoid adaptive immune resistance for colorectal cancer immunotherapy.

Apart from directly damaging tumor cells, the immune regulation abilities of chemotherapeutic drugs exhibit a great potential for tumor immunotherapy.^[^ ^7^ ^]^ Compared with antibody drugs, small molecular chemotherapeutic drugs are easy to penetrate into tumor tissues for spatiotemporal tumor management. More importantly, some antitumor drugs have been found to initiate immunogenic cell death (ICD) of colorectal cancer cells, triggering the release of damage associated molecular patterns (DAMPs) to elicit the tumor immunogenicity.^[^ ^8^ ^]^ Even so, the chemotherapy induced ICD can rarely activate systemic antitumor immunity due to the ITM and negative immune regulatory signals. Of special note, the immunosuppressive phenotype of colorectal cancer cells is dominantly affected by epigenetic gene regulation, which may provide chemo‐regulation targets to reverse the negative immune regulatory signals. Among which, the elevated expression of bromodomain and extraterminal protein 4 (BRD4) is closely associated with the epigenetic regulation of c‐Myc and PD‐L1, and c‐Myc is an important transcription factor in colorectal cancer glycolysis.^[^ ^9^ ^]^ However, such a sophisticated and synergistic mechanism is rarely investigated for chemotherapy‐enabled colorectal cancer immunotherapy.

A number of BRD4 inhibitors, including JQ1 and OTX015, had shown promising outcomes in early clinical trials.^[^ ^10^ ^]^ However, BRD4 inhibition often resulted in feedback elevation of BRD4 protein in tumor cells, leading to a weak antiproliferation activity.^[^ ^11^ ^]^ Compared with BRD4 inhibitors, proteolysis targeting chimeras (PROTACs) can degrade the protein of interest (POI) by ubiquitin‐proteasome system instead of inhibiting them, possessing some advantages of high sensitivity, selectivity, rapidity, and reversibility to regulate the protein function.^[^ ^12^ ^]^ However, most of PROTACs exhibit unfavorable physicochemical properties and poor in vivo performance for clinical translation due to the inadequate recognition with POI. Moreover, combination drug administration prefers to simultaneously elicit the tumor immunogenicity, reverse the ITM and avoid the adaptive immune resistance. In the past decades, various drug delivery systems have been fabricated for drug co‐delivery, which are also confronted with great limitations in complex procedures, cost expensive, and hard‐to‐reproduce.^[^ ^13^ ^]^ Recently, some synergistic drug pairs were found to self‐assemble into nanomedicine through intermolecular noncovalent interactions, which exhibited some appealing characteristics of minimal drug excipients, improved drug stability, and high drug loading efficiency to facilitate drug delivery.^[^ ^14^ ^]^ Even so, the complex structure of PROTACs would extensively affect the intermolecular interactions, and self‐delivery PROTACs were rarely reported.

**Scheme 1:**
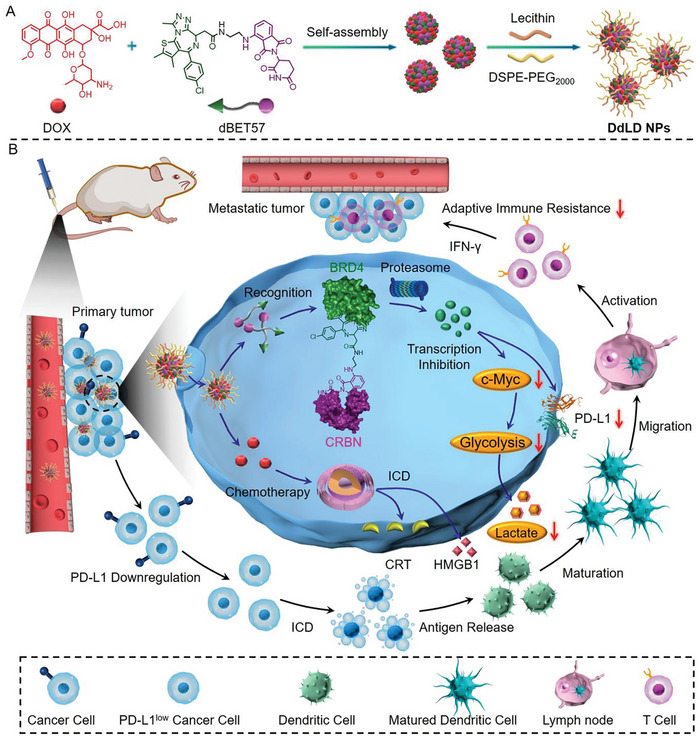
Schematic drawing of DdLD NPs for chemotherapy‐enabled colorectal cancer immunotherapy. A) Construction of DdLD NPs by the self‐assembly of DOX and dBET57 with the assistance of DSPE‐PEG_2000_ and lecithin. B) DdLD NPs could passively target the tumor site after tail vein injection. After cellular internalization, the released DOX would induce ICD response to activate antitumor immunity through antigen release. Moreover, the released dBET57 could reduce the cellular level of BRD4 protein and repress the transcriptions of PD‐L1 and c‐Myc, leading to the enhanced chemo‐immunotherapy of colorectal cancer.

In this work, the PROTAC of BRD4 (dBET57) was found to downregulate colorectal cancer glycolysis through the transcription inhibition of c‐Myc, which could also inhibit the expression of PD‐L1 to reverse immune evasion and avoid adaptive immune resistance **Scheme** **1**. To enhance the immune activation performance, self‐delivery nano‐PROTACs (designated as DdLD NPs) were further developed by self‐assembly of doxorubicin (DOX) and dBET57 with the assistance of DSPE‐PEG_2000_ Scheme 1A. Molecular dynamic simulation indicated that the assembly behavior of DOX and dBET57 was mainly driven by intermolecular noncovalent interactions, which enhanced stability and formed uniform nanoparticulated morphology. DdLD NPs could improve the intracellular delivery and tumor targeting behavior, thereby enhancing the antiproliferation and antitumor abilities through chemotherapeutic effect. Meanwhile, the chemotherapy of DdLD NPs could also initiate an ICD to release DAMPs of HMGB1 and CRT. More importantly, DdLD NPs could downregulate the glycolysis of colorectal cancer cells to reduce the lactate production, and reduce PD‐L1 expression through BRD4 degradation Scheme 1B. Taking advantages of the chemotherapy and chemo‐regulation ability, DdLD NPs could systemically activate the antitumor immunity to suppress the primary and metastatic colorectal cancer progression without inducing any systemic side effects. Such a self‐delivery strategy could promote the clinical translation of PROTACs, and the sophisticated mechanism might provide a new insight for chemotherapy‐enabled tumor immunotherapy.

## 2 Results and Discussion

### 2.1 dBET57‐Mediated Downregulation of PD‐L1 and c‐Myc

**Figure 1:**
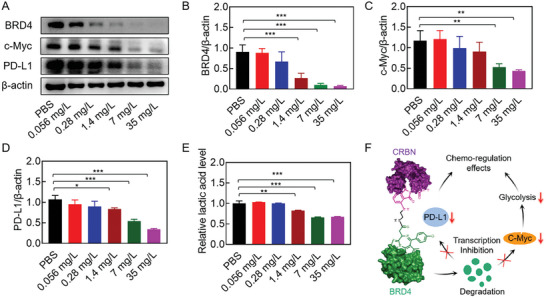
The repression of PD‐L1 and c‐Myc transcriptions through dBET57‐mediated BRD4 degradation. A) Representative immunoblots and quantitative analyses of B) BRD4, C) C‐Myc, and D) PD‐L1 (*n* = 3). E) The lactic acid content in CT26 cell culture medium after treatment with different concentrations of dBET57 (*n* = 3). F) Schematic drawing of dBET57‐mediated chemo‐regulation effects. The numerical calculation results were presented as means ± SD. Statistical analysis was performed by one‐way ANOVA. ^*^p < 0.05, ^**^p < 0.01, and ^***^p < 0.001.

We first evaluated whether dBET57 could induce BRD4 degradation, thus inhibiting the PD‐L1 and c‐Myc transcription. As displayed in **Figure** **1**A, dBET57‐mediated BRD4 degradation was found to be concentration dependent. After being treated with gradient concentrations of dBET57, the protein expression of BRD4 in CT26 cells was rapidly depleted. In particular, nearly 70.5% of BRD4 protein had been depleted at the concentration of 1.4 mg L^−1^ (Figure 1B), indicating the high protein degradation efficiency of dBET57. Moreover, the degradation of BRD4 inhibited the transcriptions of PD‐L1 and c‐Myc, therefore reducing their protein expressions (Figure 1C,D). c‐Myc was reported as a “master regulator” of tumor glycolysis, which could regulate the lactate production. As shown in Figure 1E, the lactate levels in culture medium were significantly reduced after dBET57 treatment. All above results indicated the chemo‐regulation effect of dBET57, which would be of great benefit for chemotherapy‐induced immune response (Figure 1F).

### 2.2 Preparation and Characterization of DdLD NPs

**Figure 2:**
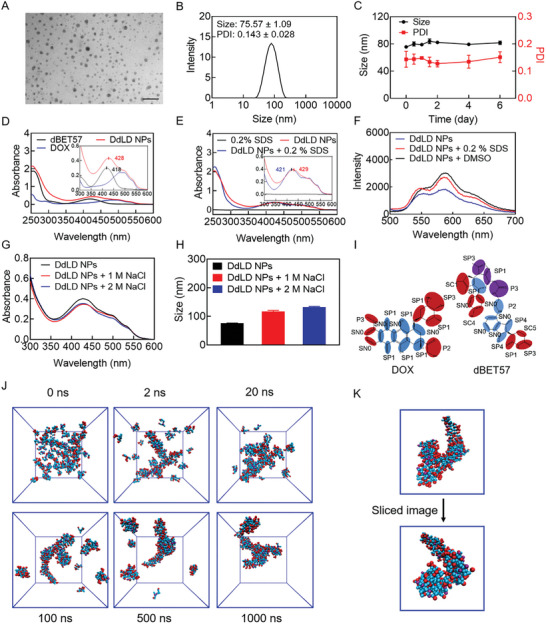
Characterizations of DdLD NPs. A) TEM image of DdLD NPs. Scale bar: 500 µm. B) Particle size histogram of DdLD NPs. C) Size and PDI value changes of DdLD NPs in six days (*n* = 3). D) The complete and enlarged absorbance spectra of dBET57, DOX, and DdLD NPs. E) The complete and enlarged absorbance spectra of DdLD NPs before and after treatment with 0.2% SDS. F) Fluorescence spectra of DdLD NPs after various treatments. G) Absorbance spectra and H) particle size values of DdLD NPs before and after treatment with 1 or 2 m NaCl solution (*n* = 3). I) Coarse‐grained models of DOX and dBET57. J) CGMD simulations on the self‐assembly of DOX and dBET57 in solution. K) The sliced image of one DOX‐dBET57 nanoparticle. Water beads are omitted for clarity. The numerical calculation results were presented as means ± SD.

Given the advantages of dBET57 for downregulation of PD‐L1and c‐Myc, we next sought to develop self‐delivery nanomedicines based on dBET57 and DOX. DOX was reported to interact with hydrophobic drugs such as 10‐hydroxycamptothecin, celastol, and chlorin e6 by hydrophobic and π–π interactions.^[^ ^15^ ^]^ To prepare ideal nanomedicine, different molar ratios of DOX and dBET57 were added into pure water. TEM images showed that the nanomedicine prepared at the ratio of 3:2 exhibited relatively integrated and uniform morphology (Figure S1, Supporting Information). Therefore, the ratio of 3:2 was selected for further studies. To prolong the circulation time of self‐assembled nanomedicines in vivo, DSPE‐PEG_2000_ and lecithin were added to increase the stability of nanomedicines. As showed in **Figure** **2**A,B, DdLD NPs exhibited uniform spherical morphology with an average size of 75.57 nm. Moreover, the size and PDI values of DdLD NPs had remained stable in physiological conditions (Figure 2C and Figure S2, Supporting Information), which would help for biomedical application. By using high performance liquid chromatography (HPLC), the loading rates of DOX and dBET57 in DdLD NPs were verified to be 23.6% and 35.3%, while the entrapment efficiencies were 21.1% and 36.8%, respectively (Figure S3, Supporting Information). The high drug‐loading efficiency would facilitate drug delivery and reduce ingredients‐induced potential side effects. The self‐assembly mechanism was then investigated by recording the UV–vis spectra of DdLD NPs in different incubation conditions. The spectrum of DdLD NPs possessed both characteristic absorptions of DOX and dBET57. Meanwhile, the characteristic absorption wavelength of DdLD NPs had a red‐shift compared with dBET57 (Figure 2D). The results indicated that DOX and dBET57 could self‐assemble into nanomedicine. When treated with 0.2% SDS, there was a 8 nm blue‐shift in the absorption spectrum of DdLD NPs (Figure 2E) and the fluorescence intensity of DOX increased (Figure 2F), which confirmed hydrophobic and π–π interactions involved in the self‐assembly process. NaCl was used to shield intermolecular electrostatic interaction. The absorbance spectrum and particle size of DdLD NPs changed a lot after incubation with NaCl (Figure 2G,H), indicating that the electrostatic interaction also played an important role in driving the self‐assembly of DOX and dBET57. Subsequently, the molecular dynamics simulation was also performed to explore the self‐assembly process of DOX and dBET57 (Figure 2I–K). The intermolecular forces between DOX and dBET57 promoted their aggregation in aqueous solution. The hydrophilic functional groups such as amino group and hydroxyl group were distributed throughout the surface of nanoparticles. Moreover, DOX would interact with dBET57 between nonpolar groups through hydrophobic and π–π interactions, and both drugs could form electrostatic interaction with water molecules to maintain structural stability. Above all, it could be speculated that hydrophobic, electrostatic, and π–π interactions were the main driving forces in the self‐assembly process of DOX and dBET57.

### 2.3 Cytotoxicity and ICD Induction of DdLD NPs In Vitro

**Figure 3:**
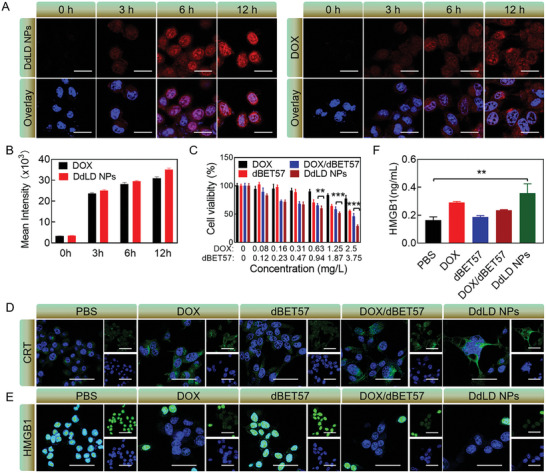
The antitumor effect of DdLD NPs. A) CLSM images of CT26 cells‐treated with DOX or DdLD NPs at different time points. Scale bar: 20 µm. B) The mean fluorescence intensity of DOX in CT26 cells at different time points (*n* = 3). C) Cell viability of CT26 cells after being treated with different formulas (*n* = 5). Immunofluorescence images of D) CRT and E) HMGB1 in CT26 cells. Scale bar: 50 µm. F) The HMGB1 content of CT26 cell culture medium (*n* = 3). The numerical calculation results were presented as means ± SD. Statistical analysis was performed by Student's *t*‐test. ^**^p < 0.01 and ^***^p < 0.001.

Prior to evaluating the cytotoxicity of DdLD NPs, the cellular uptake behavior was investigated by CLSM and flow cytometry. CT26 cells incubated with DOX were set as the control. Fluorescence images showed that the fluorescent intensity of DOX in both DdLD NPs and DOX groups gradually increased as time went on (**Figure** **3**A). The nuclear enrichment of DOX was observed in DdLD NPs group (Figure S4, Supporting Information), which would help to intercalate DNA to kill tumor cells. Moreover, the red fluorescence in DdLD NPs‐treated cells was stronger than that of DOX‐treated cells. A similar result was found in flow cytometry analysis (Figure 3B), suggesting a time‐dependent enhancement of cellular uptake upon DdLD NPs treatment.

Next, CT26 cells were treated with different therapeutic agents for MTT assay to test cytotoxicity (Figure 3C). DOX and dBET57 showed relatively low toxicity to CT26 cells that the cell viabilities kept at 77.3% and 55.6%, respectively. The mixture of DOX and dBET57 exhibited a higher concentration‐dependent cytotoxicity. However, under the same dosages of DOX and dBET57, DdLD NPs achieved the best efficiency in growth inhibition, which might be attributed to the enhanced cellular internalization of nanomedicine. Besides, the cytotoxicity of DdLD NPs was also detected toward normal cells. More than 60% of the mouse embryonic fibroblast cells (3T3) were alive at the maximum dose of DdLD NPs (Figure S5, Supporting Information), indicating a relatively low toxicity of DdLD NPs against normal cells Scheme.

Additionally, the DdLD NPs‐induced ICD effect was detected in vitro. The CRT exposure and HMGB1 release were two important signals of ICD response.^[^ ^16^ ^]^ Therefore, the immunofluorescence staining was conducted to analyze the expressions of CRT and HMGB1 in CT26 cells. CLSM images showed the increased green fluorescence intensity in DOX, DOX/dBET57, and DdLD NPs‐treated cells, indicating the exposure of CRT on membrane (Figure 3D). Moreover, the green fluorescence of HMGB1 protein was widely observed in PBS and dBET57‐treated cells, however, they were clearly decreased in DOX, DOX/dBET57, and DdLD‐treated groups, demonstrating the extracellular efflux of HMGB1 (Figure 3E). The extracellular efflux of HMGB1 was also detected by enzyme‐linked immunosorbent assay (ELISA). As shown in Figure 3F, there was about 2.23‐fold increased HMGB1 release in DdLD NPs group compared to that of PBS group. Apparently, DdLD NPs caused the most release of HMGB1 and exposure of CRT, proving the strong ICD effect induced by DdLD NPs.

### 2.4 DdLD NPs‐Mediated Downregulation of PD‐L1 and c‐Myc

**Figure 4:**
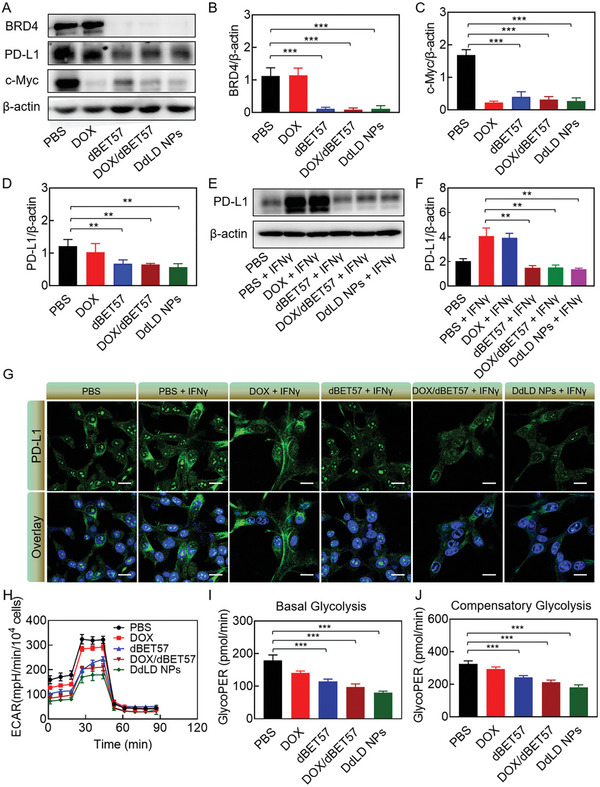
The repression of PD‐L1 and c‐Myc transcriptions through DdLD NPs‐mediated BRD4 degradation. A) Representative immunoblots and quantitative analyses of B) BRD4, C) C‐Myc and D) PD‐L1 (*n* = 3). E) Representative immunoblots and quantitative analysis of F) PD‐L1 (*n* = 3). G) CLSM images of PD‐L1 in CT26 cells after treatment with different formulas. Scale bar: 20 µm. H) The ECAR curve of CT26 cells after treatment with different formulas (*n* = 3). I) The glycolytic PER of basal glycolysis and J) compensatory glycolysis in CT26 cells after treatment with different formulas (*n* = 3). The numerical calculation results were presented as means ± SD. Statistical analysis was performed by one‐way ANOVA. ^**^p < 0.01 and ^***^p < 0.001.

In order to further confirm the effect of DdLD NPs on CT26 cells, western blotting analysis was conducted to evaluate the expressions of BRD4, PD‐L1, and c‐Myc proteins. As shown in **Figure** **4**A, compared with PBS group, dBET57, dBET57/DOX, and DdLD NPs significantly decreased the protein expression of BRD4, indicating the ability of DdLD NPs for regulating the BRD4 protein level. Corresponding quantitative results showed that above 90% of BRD4 protein was depleted after incubation with dBET57, dBET57/DOX, and DdLD NPs (Figure 4B). Meanwhile, PD‐L1 and c‐Myc proteins were significantly downregulated owing to the depletion of BRD4 protein (Figure 4C,D). However, no statistically significant difference was observed between the groups of dBET57, dBET57/DOX, and DdLD NPs, which might be ascribed to the high protein degradation efficiency of dBET57. Interestingly, DOX‐treated cells also showed an obvious downregulation of c‐Myc protein. As we know, c‐Myc had a pivotal role in tumor growth, differentiation, and apoptosis, whose abnormal expression was closely associated with some malignant tumors.^[^ ^17^ ^]^ DOX was an inhibitor of DNA‐topoisomerase II, which could also inhibit c‐Myc expression and promote cell apoptosis. Mechanically speaking, DOX could inactivate JAK‐STAT3 pathway by suppressing the phosphorylation of STAT3 and JAK, thereby decreasing the expression of c‐Myc to induce cell apoptosis.^[^ ^18^ ^]^

DOX could induce ICD response for antitumor immunity activation. However, tumor‐infiltrating CTLs usually killed tumor cells by secreting large amounts of IFN‐γ, which would reversely inactivate CTLs by upregulating PD‐L1 protein. Therefore, the expression of PD‐L1 protein in IFN‐γ‐treated CT26 cells was evaluated by western blotting analysis. As shown in Figure 4E,F, IFN‐γ‐treated CT26 cells showed 1.99‐fold upregulation of PD‐L1 expression compared to that of PBS‐treated cells. After treatment with dBET57, dBET57/DOX, or DdLD NPs, the cells exhibited significantly lower protein expression of PD‐L1 than those treated with IFN‐γ. To further confirm this, the immunofluorescence staining was also conducted (Figure 4G). Obviously, dBET57, dBET57/DOX, and DdLD NPs inhibited the expression of PD‐L1 that IFN‐γ‐pretreated CT26 cells presented weaker immunofluorescence signals. These results indicated that DdLD NPs had the ability to reverse IFN‐γ‐inducible immune resistance.

To explore whether downregulation of c‐Myc could result in the inhibition of tumor glycolysis, the glycolysis rate of CT26 cells was detected after treatment with various agents (Figure 4H). The extracellular acidification rate (ECAR) curves of dBET57 and dBET57/DOX‐treated cells were lower than that of PBS‐treated cells, indicating an inhibition of glycolytic function and decrease in lactate production. DdLD NPs exhibited the best efficiency in glycolysis inhibition, which might be attributed to the enhanced cellular internalization of DdLD NPs. Moreover, the glycolytic function was also evaluated by proton efflux rate (PER) detection and consistent results were found. dBET57 and dBET57/DOX were able to impair glycolysis that the glycolytic PER (glycoPER) was significantly decreased in both basal and compensatory glycolysis (Figure 4I,J). In DdLD NPs‐treated cells, the glycoPER was the lowest that the basal and compensatory glycolysis decreased over 55% and 45%, respectively. Without any doubt, DdLD NPs could effectively hinder extracellular acidification and proton efflux. In light of the results above, DdLD NPs could significantly inhibit PD‐L1 and c‐Myc expressions by inducing BRD4 degradation, which confirmed efficient chemo‐regulation effects of DdLD NPs.

### 2.5 In Vivo Antitumor Effect of DdLD NPs

**Figure 5:**
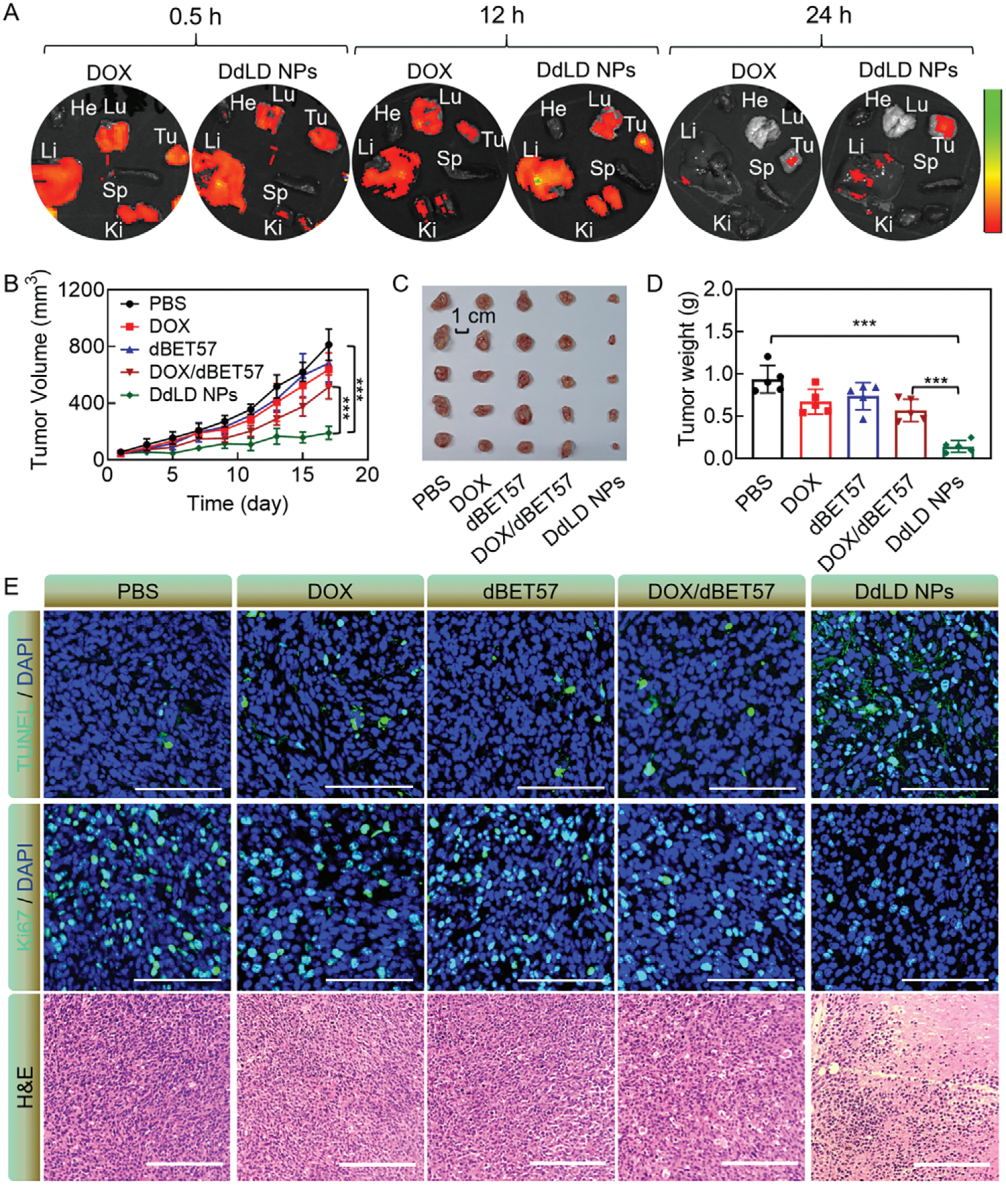
A) Fluorescence images of isolated tissues from CT26 tumor‐bearing mice following intravenous injection of DdLD NPs or DOX. B) The tumor volume changes, C) tumor image, and D) tumor weights of CT26 tumor‐bearing mice following treatment with PBS, DOX, dBET57, DOX/dBET57, and DdLD NPs (*n* = 5). E) TUNEL, Ki67, and H&E staining of tumor tissues following treatment with PBS, DOX, dBET57, DOX/dBET57, and DdLD NPs. Scale bar: 100 µm. The numerical calculation results were presented as means ± SD. Statistical analysis was performed by one‐way ANOVA. ^***^p < 0.001.

The efficient chemotherapeutic and chemo‐regulation effects of DdLD NPs inspired us to further evaluate its antitumor effect in vivo. The selective accumulation of DdLD NPs was first studied using CT26 tumor‐bearing mice after intravenous injection. At the indicated time points, the major organs and tumor tissues were extracted and collected for ex vivo fluorescence imaging (**Figure** **5**A). The fluorescence signal was mainly localized in liver, lung, kidney, and tumor tissues. As time went by, the fluorescence intensity of DdLD NPs‐treated tumor tissue was stronger than that of DOX‐treated tumor tissue, which was also confirmed by the quantitative fluorescence analysis of isolated tissues (Figure S6, Supporting Information). Moreover, the fluorescence intensity obviously decreased as a function of time in these organs except for the tumor tissue, demonstrating a good tumor retention ability of DdLD NPs through passive targeting behavior.

Subsequently, the antitumor study was performed to validate whether DdLD NPs could suppress the tumor growth. After intravenous injection with different therapeutic agents, the tumor volumes of CT26 tumor‐bearing mice were regularly recorded (Figure 5B). The tumor volumes in PBS group increased sharply. DOX and dBET57 could induce a slight reduction of tumor volumes, which might result from the fact of DOX and dBET57‐induced cytotoxicity. The mixture of DOX and dBET57 showed a relatively good therapeutic effect. In contrast, DdLD NPs exhibited the highest efficiency on tumor inhibition at the same dosages of DOX and dBET57, demonstrating the superiority of nanomedicine in tumor therapy and the synergistic effect of DOX and dBET57. Consistent results were also found in tumor image and weight analyses (Figure 5C,D). DdLD NPs reduced the tumor weight by 84.6%, showing an overwhelming advantage over other groups. Afterward, the tumor tissues were collected for H&E, TUNEL, and Ki67 staining, respectively (Figure 5E). The H&E staining showed that DdLD NPs group had the maximum apoptotic or necrosis tumor cells without nuclei. The strongest TUNEL signal and highest positive rate (12.9%) were observed in DdLD NPs group (Figure S7, Supporting Information), indicating the most apoptotic cells in tumor tissue. Ki67 immuno‐staining showed that DdLD NPs‐treated tumor tissue had the weakest Ki67 signal and lowest positive rate (6.2%) (Figure S8, Supporting Information), suggesting that DdLD NPs could significantly inhibit tumor cell proliferation. Moreover, the body weights of the mice were recorded every 2 days (Figure S9, Supporting Information), which showed almost no fluctuations. All above results demonstrated that DdLD NPs could greatly repress the tumor growth without remarkable system toxicity.

### 2.6 Antitumor Immune Activation of DdLD NPs

**Figure 6:**
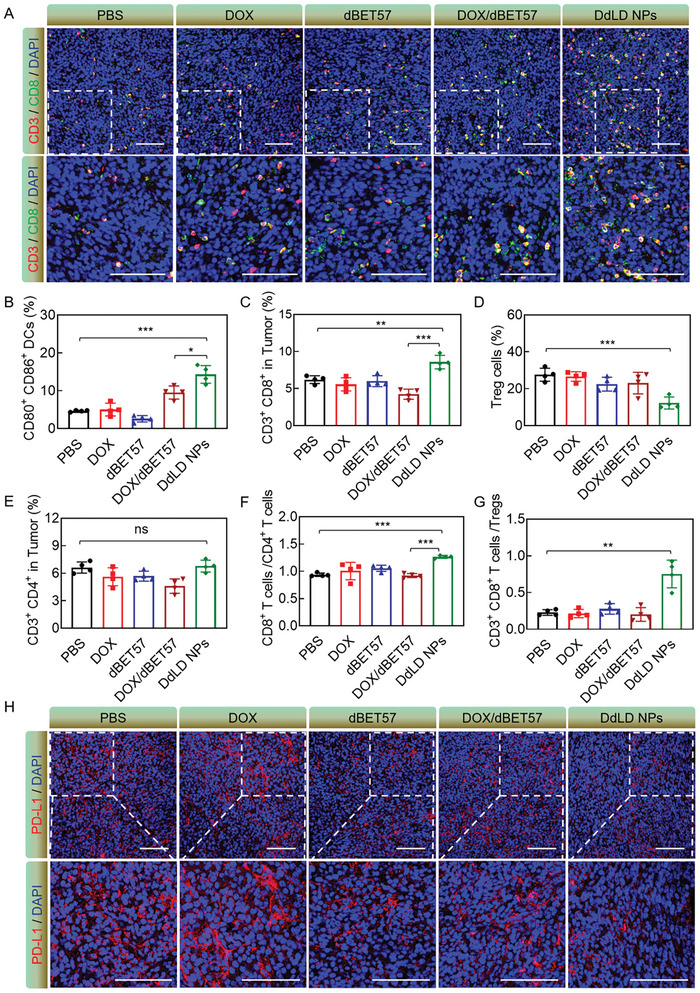
A) CD3 and CD8 immunofluorescence staining of tumor tissues. Scale bar: 100 µm. Percentages of B) CD80^+^ CD86^+^ DCs, C) CD3^+^ CD8^+^ T cells, D) Treg cells and E) CD3^+^ CD4^+^ T cells in tumor tissues (*n* = 4). F) The ratio of CD3^+^ CD8^+^ T cells and CD3^+^ CD4^+^ T cells in tumor tissues. G) The ratio of CD3^+^ CD8^+^ T cells and Treg cells in tumor tissues. H) PD‐L1 immunofluorescence staining of tumor tissues. Scale bar: 100 µm. The numerical calculation results were presented as means ± SD. Statistical analysis was performed by one‐way ANOVA or Student's *t*‐test. ^*^p < 0.05, ^**^p < 0.01, and ^***^p < 0.001.

To investigate whether DdLD NPs could activate antitumor immunity, the infiltrating T cells of tumor tissues were detected by CD3 and CD8 immunofluorescence staining (**Figure** **6**A). Compared with PBS group, DOX, dBET57, and DOX/dBET57 caused a negligible influence on CD3^+^CD8^+^ T cells infiltration that the images exhibited similar immunofluorescence intensity as PBS group. But the tumor tissues treated by DdLD NPs had the strongest red and green immunofluorescence signal, indicating the most infiltrated CD3^+^ CD8^+^ T cells. To confirm it, immune cell populations of tumor tissue were detected by flow cytometry (Figure S10–S12, Supporting Information). As shown in Figure 6B, C, DdLD NPs increased the percentages of CD80^+^ CD86^+^ dendritic cells (DCs) and CD3^+^ CD8^+^ T cells in tumor tissues. Of special note, the proportions of CD80^+^ CD86^+^ DCs and CD3^+^ CD8^+^ T cells were 9.5% and 4.2% in DOX/dBET57 group, while the proportions of these cells were 14.3% and 8.6% in DdLD NPs group. Moreover, although DdLD NPs had no distinct effect on CD3^+^ CD4^+^ T cells, the ratio of CD3^+^ CD8^+^ T cells to CD3^+^ CD4^+^ T cells in DdLD NPs group was 1.37‐fold higher than that of in DOX/dBET57 group (Figure 6E,F). Such obvious growths should be mainly ascribed to the improved drug delivery efficiency and co‐localization of DOX and dBET57 in tumor tissue by nanomedicine. Besides, the mice‐treated with DdLD NPs showed the lowest proportion of CD3^+^ CD4^+^ FOXP3^+^ regulatory T cells (Tregs), and the ratio of CD3^+^ CD8^+^ T cells to Tregs in DdLD NPs group was 3.63‐fold and 3.20‐fold higher than that of in DOX/dBET57 and PBS groups (Figure 6D,G). Tregs would blunt the activity of CTLs and promote ITM. Therefore, the reduction of Tregs was of great benefit for activating the antitumor immune response. The PD‐L1 expression on the tumor cells could bind to PD‐1 expressed on the surface of CTLs, leading to the inactivation of CTLs. In order to evaluate the PD‐L1 expression on tumor tissues, the PD‐L1 immunofluorescence staining was carried out (Figure 6H). It was important that apart from dBET57, DdLD NPs also had the ability to decrease the PD‐L1 expression of tumor cells, which would help to reverse immune evasion and avoid adaptive immune resistance. These results confirmed that DdLD NPs was capable of relieving the ITM to promote the activation of antitumor immunity.

### 2.7 Metastatic Tumor Inhibition

**Figure 7:**
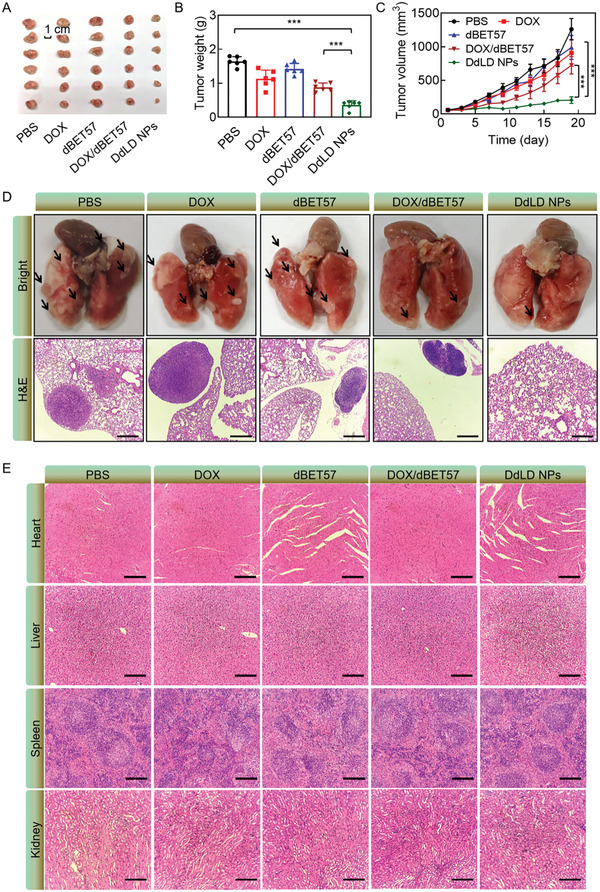
A) The image and B) mean weights of tumor tissues. C) The tumor growth curves of CT26 tumor‐bearing mice after treatment with various therapeutic agents (*n* = 6). D) The images and H&E staining of lung tissues. Scale bar: 500 µm. E) The H&E staining of heart, liver, spleen, and kidney. Scale bar: 100 µm. The numerical calculation results were presented as means ± SD. Statistical analysis was performed by one‐way ANOVA. ^***^p < 0.001.

Then, we evaluated the therapeutic effect of DdLD NPs on metastatic tumors. CT26 cells were injected into tail vein to construct the metastatic tumor model. After treatment with various therapeutic agents, the primary tumor volumes were recorded. The tumor growth curves showed that DdLD NPs had the best antitumor effect (**Figure** **7**A). Moreover, the tumor tissues were dissected to photograph (Figure 7B) and weigh (Figure 7C) at the end of treatment. The tumor tissues in DdLD NPs exhibited the smallest volume and lightest weight, which showed the effective suppression of tumor growth. Additionally, the lung tissues were collected for photographing and H&E staining. As displayed in Figure 7D, DdLD NPs was capable of inhibiting the lung metastasis. Corresponding quantitative results showed that although all treatment groups displayed lung metastases (Figure S13, Supporting Information), the number of average lung metastatic loci was the least in DdLD NPs group, indicating an effective metastasis suppression of DdLD NPs‐mediated chemotherapy‐enabled immunotherapy. The main organs were also collected for H&E staining (Figure 7E). Although the therapeutic agent had nonspecific distribution in normal tissues, there was no obvious morphological abnormity in heart, kidney, liver, and spleen. The recorded body weights showed almost no fluctuations (Figure S14, Supporting Information), suggesting low system toxicities of those agents. At the end of treatments, the serums were collected from experimental mice for biochemical analysis. The indicators of liver (ALT and AST) and kidney (UA and BUN) functions were all within normal ranges (Figure S15, Supporting Information), indicating that those agents had no apparent damage to liver and kidney. All above results demonstrated that DdLD NPs could greatly repress lung metastasis with little side effects to normal tissues.

## 3 Conclusion

In summary, we had developed self‐delivery nano‐PROTACs (designated as DdLD NPs) to realize chemotherapy‐enabled colorectal cancer immunotherapy by inhibiting tumor glycolysis and avoiding adaptive immune resistance. The DdLD NPs‐based antitumor strategy reported here presented some advantages in novelty. First, DdLD NPs were constructed by the self‐assembly of DOX and dBET57, which could be easy for preparation and achieved drugs co‐delivery without the assistance of carriers. Second, the chemotherapy of DdLD NPs could efficiently destroy colorectal cancer cells to trigger a robust ICD. Moreover, the chemo‐regulation effects of DdLD NPs could inhibit colorectal cancer glycolysis to reduce the lactate production, which downregulated PD‐L1 expression through BRD4 degradation. Taking advantages of the chemotherapy and chemo‐regulation ability, DdLD NPs could systemically activate the antitumor immunity to suppress the primary and metastatic colorectal cancer progression. Third, the application of BRD4‐degrader dBET57 could regulate the protein function with a more sensitive and rapid modality, which exhibited a higher therapeutic efficiency than that of small‐molecule drugs. Therefore, such self‐delivery nano‐PROTACs might provide a new insight for chemotherapy‐enabled tumor immunotherapy.

## 4 Experimental Section

### Formulation of DdLD NPs

To prepare DdLD NPs, the mixture solution was first prepared containing 69.9 µL of dBET57 stock solution (10 mg mL^−1^, DMSO), 20 µL of lecthin (5 mg mL^−1^, DMSO), 40 µL of DSPE‐PEG_2000_ (5 mg mL^−1^, DMSO) and 81.5 µL of DOX aqueous solution (10 mg mL^−1^). The mixture was then dropped into 2 mL ultrapure water. After being stirred for 0.5 h, the above solution was stocked in darkness overnight. Next, the solution was dialyzed to remove free drugs. Finally, DdLD NPs were collected for further investigation.

### Characterization of DdLD NPs

The appearance and size value of DdLD NPs were detected by a transmission electron microscope (TEM) and particle size analyzer, respectively. To investigate the intermolecular force in the self‐assembly, DdLD NPs were premixed with different concentrations of SDS or NaCl. After incubation for 30 min, the absorption spectra of these solutions were detected by using a UV spectrophotometer. The self‐assembly process of DOX and dBET57 was validated by coarse‐grained molecular dynamics (CGMD) simulation. Moreover, the drug loading rate and entrapment efficiency of DOX and dBET57 were measured using HPLC after dissolving DdLD NPs in DMSO. The mobile phase consisted of 0.1% trifluoroacetic acid in water (40%) and acetonitrile (60%). Drug loading rate (%) = (Weight of DOX or dBET57 loaded) / Weight of DdLD NPs × 100%. Entrapment efficiency (%) = (Weight of DOX or dBET57 loaded) / Weight of total DOX or dBET57 × 100%.

### Cellular Uptake Assay

CT26 cells were seeded in confocal dish and cultured with DOX (5 mg L^−1^) and DdLD NPs (21.2 mg L^−1^). After incubation for 0, 3, 6, or 12 h, the cellular nuclei were stained with hoechst 33342. The cellular uptake efficiency was analyzed by using a CLSM.

### Calreticulin (CRT) and HMGB1 Immunofluorescence Staining

CT26 cells were seeded in confocal dish. After being treated with PBS, DOX (5 mg L^−1^), dBET57 (7.5 mg L^−1^), DOX/dBET57 (12.5 mg L^−1^), or DdLD NPs (21.2 mg L^−1^) for 12 h, CT26 cells were fixed and permeabilized. After that, the cells were incubated with CRT or HMGB1 primary antibody overnight at 4 °C. The secondary antibody was then used to visualize the protein expression of CRT or HMGB1 in CT26 cells.

### Cytotoxicity Assay

CT26 cells were cultured in 96‐well plates. The gradient concentrations of DOX, dBET57, DOX/dBET57, or DdLD NPs were added into the wells. After incubation for 24 h, the cell viability of CT26 cells was detected by standard MTT assay. As the control, the toxicity of DdLD NPs to normal cells was also detected on mouse embryonic fibroblast cells of 3T3.

### Western Blotting Analysis

CT26 cells were seeded in six‐well plates. After being treated and incubated for 24 h, the cells were lysed and collected in microcentrifuge tubes. After centrifugation, cellular proteins were separated and transferred onto PVDF membranes. The membranes were then blocked by 5% skim milk. After incubation with BRD4, c‐Myc, PD‐L1, or β‐actin primary antibody overnight, the membranes were followed by incubation with secondary antibody. Finally, the expression of these proteins was visualized by dropping chemiluminescent substrate onto the membrances.

### Cellular Glycolysis Rate Measurement

CT26 cells were seeded in seahorse XF 24‐well plates for overnight attachment. They were then incubated with PBS, DOX (5 mg L^−1^), dBET57 (7.5 mg L^−1^), DOX/dBET57 (12.5 mg L^−1^), or DdLD NPs (21.2 mg L^−1^) for 10 h. After that, the cells were washed with seahorse XF base medium for three times and then incubated in a CO_2_‐free incubator. After being treated with rotenone/antimycin A and 2‐deoxyglucose, the glycolysis rate of CT26 cells was detected by using a seahorse XF analyzer.

### Ex Vivo Distribution of DdLD NPs

In this study, all the animal experiments were performed in accordance with the guidelines of Institutional Animal Care and Use Committee (IACUC) of Animal Experiment Center of Guangzhou Medical University (Guangzhou, China). To detect the distribution of DdLD NPs, DOX (1.33 mg kg^−1^) or DdLD NPs (5.64 mg kg^−1^) was intravenously injected to CT26 tumor‐bearing BALB/c mice. At the indicated time points, the organs were collected for fluorescence imaging.

### Antitumor Study and Antitumor Immunity Assessment of DdLD NPs In Vivo

To assess the antitumor activity of DdLD NPs, CT26 tumor‐bearing BALB/c mice were constructed by subcutaneously injecting CT26 cells (1 × 10^6^) into the right hind leg. After being randomly divided into five groups, the CT26 tumor‐bearing mice were intravenously injected with PBS, DOX, dBET57, DOX/dBET5,7 or DdLD NPs (at equivalent DOX dose of 1.33 mg kg^−1^ and dBET57 dose of 2 mg kg^−1^). The mice received treatments every two days for a total of five times. The tumor volume and weight were regularly recorded during the treatments. At the end of treatments, the tumor tissues were collected and fixed for further H&E staining.

To evaluate the immune activation of DdLD NPs, CT26 tumor‐bearing BALB/c mice were intravenously injected with various therapeutic agents. After five times of treatments, the tumor tissues were collected and digested with digestion buffer containing collagenase IV and hyaluronidase. The single cell suspension was obtained and then stained with various antibodies for flow cytometry detection.

### Metastatic Tumor Inhibition

In order to establish metastatic tumor model, the CT26 tumor‐bearing BALB/c mice were intravenously injected with CT26 cells (1 × 10^5^). After being divided into five groups, the mice were administrated with PBS, DOX, dBET57, DOX/dBET57, or DdLD NPs. The therapeutic agents were injected via tail vein at equivalent DOX dose of 1.33 mg kg^−1^ and dBET57 dose of 2 mg kg^−1^. The mice received treatments every two days for a total of five times. The tumor volume and weight were regularly recorded during the treatments. At the end of treatments, the tumor tissues and organs were collected and fixed for further H&E staining.

### Statistical Analysis

The numerical calculation results were presented as means ± SD through at least three independent samples. Student's *t*‐test (between two groups) and One‐way analysis of variance (ANOVA) (within multiple groups) were used for statistical analysis. All statistical analyses were conducted by the GraphPad Prism 8.0 software. *p < 0.05* was defined to be statistically significant.

## Conflict of Interest

The authors declare no conflict of interest.

## Supporting information

Supporting Information

## Data Availability

The data that support the findings of this study are available from the corresponding author upon reasonable request.
