# Supplementary material for: Chemotherapy‐Enabled Colorectal Cancer Immunotherapy of Self‐Delivery Nano‐PROTACs by Inhibiting Tumor Glycolysis and Avoiding Adaptive Immune Resistance
Source: Adv Sci (Weinh). 2024 Jan 18;11(15):2309204. doi: 10.1002/advs.202309204 (PMC11022706; doi:10.1002/advs.202309204)
Supplement: Supplementary file 1 — Supporting Information [file ADVS-11-2309204-s001.pdf]

ADVANCED SCIENCE
Open Access

# Supporting Information

for *Adv. Sci.*, DOI 10.1002/advs.202309204

Chemotherapy-Enabled Colorectal Cancer Immunotherapy of Self-Delivery Nano-PROTACs by Inhibiting Tumor Glycolysis and Avoiding Adaptive Immune Resistance

*Lin-Ping Zhao, Rong-Rong Zheng, Xiao-Na Rao, Chu-Yu Huang, Hang-Yu Zhou, Xi-Yong Yu, Xue-Yan Jiang and Shi-Ying Li**

# Supporting Information

## Chemotherapy-Enabled Colorectal Cancer Immunotherapy of Self-Delivery Nano-PROTACs by Inhibiting Tumor Glycolysis and Avoiding Adaptive Immune Resistance

*Lin-Ping Zhao†, Rong-Rong Zheng†, Xiao-Na Rao†, Chu-Yu Huang, Hang-Yu Zhou, Xi-Yong Yu, Xue-Yan Jiang, Shi-Ying Li**

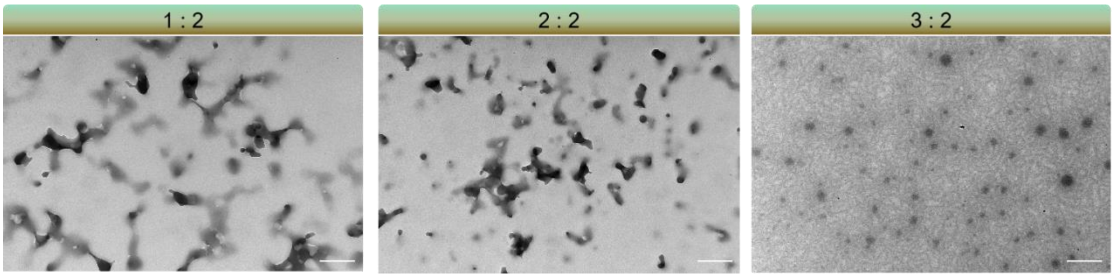


**Figure S1.** TEM images of nanomedicines prepared at various molar ratios. Scale bar: 500 μm.

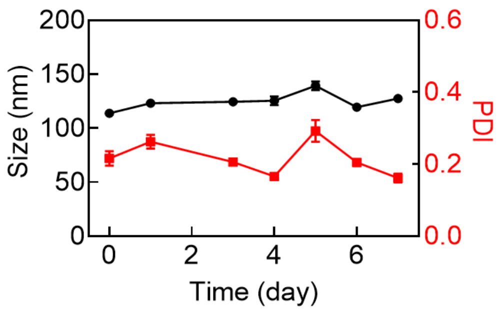


**Figure S2.** Stability of DdLD NPs in PBS containing 10 % FBS (n = 3). The numerical calculation results were presented as means ± SD.

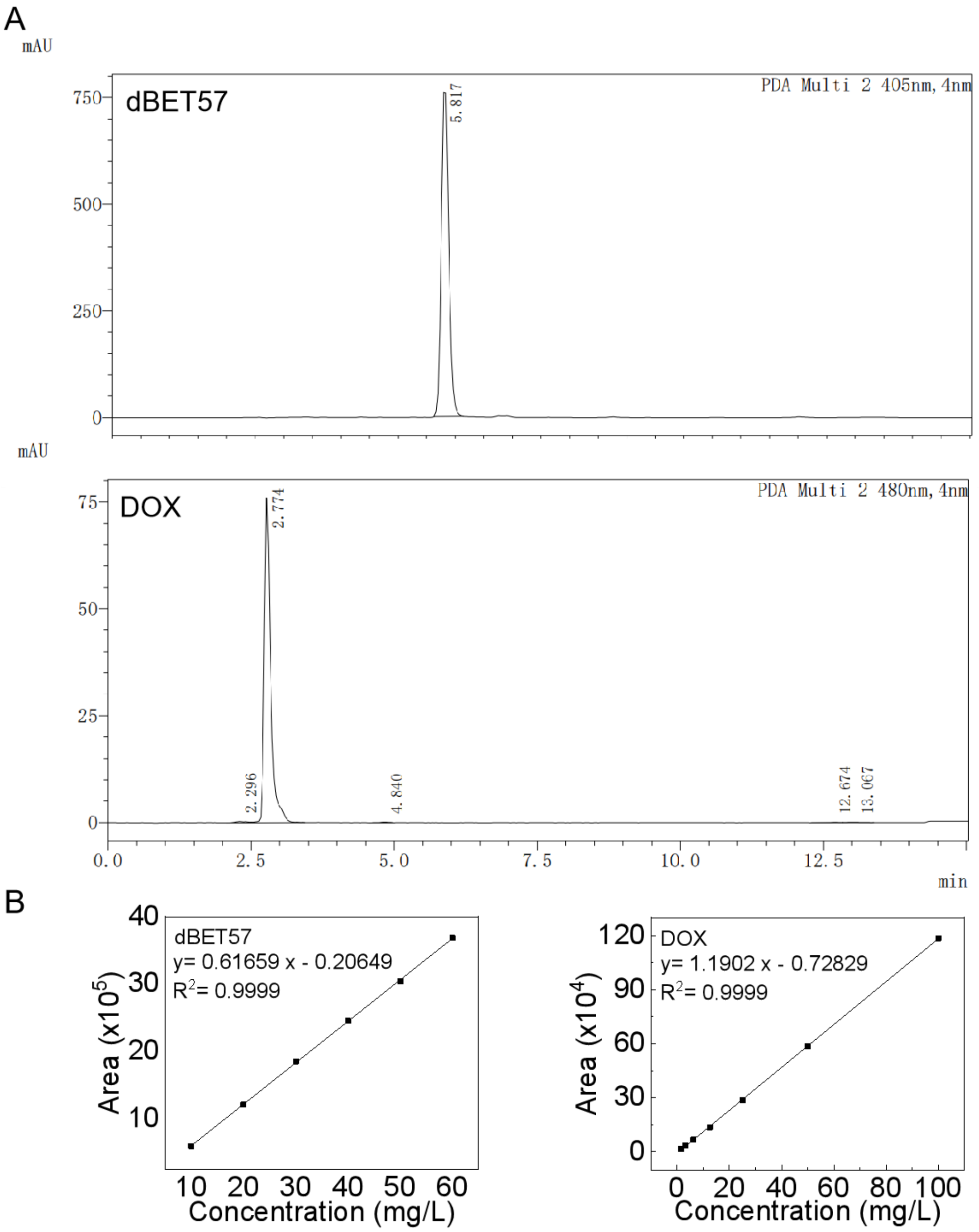


**Figure S3.** (A) The retention time of dBET57 and DOX in HPLC. (B) Standard curves of dBET57 and DOX.

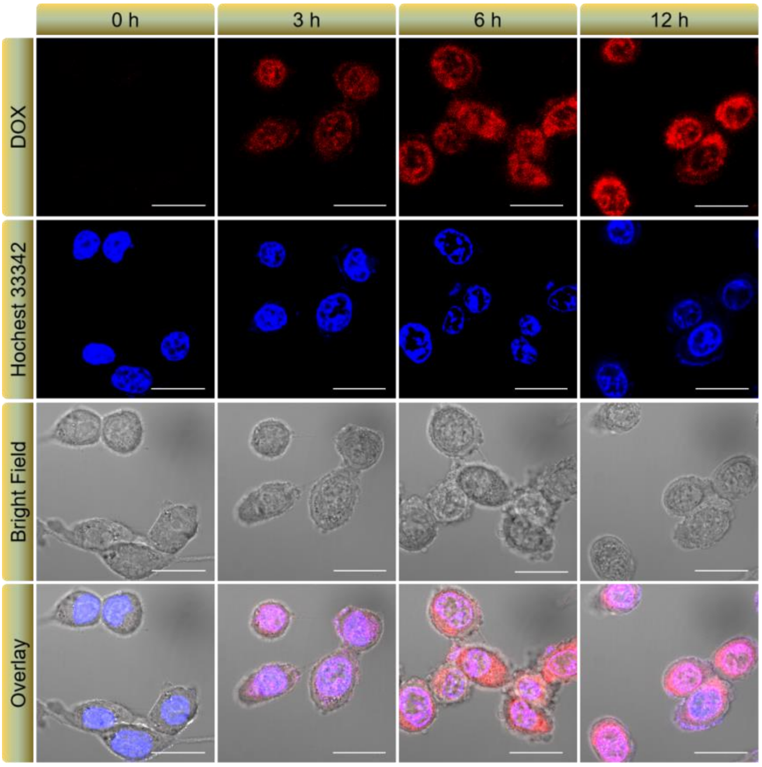


**Figure S4.** CLSM images of CT26 cells-treated with DdLD NPs at different time points. Scale bar: 20 μm.

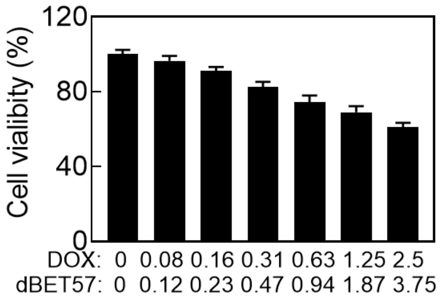


**Figure S5.** The cytotoxicity of DdLD NPs towards 3T3 cells (n = 5). The numerical calculation results were presented as means ± SD.

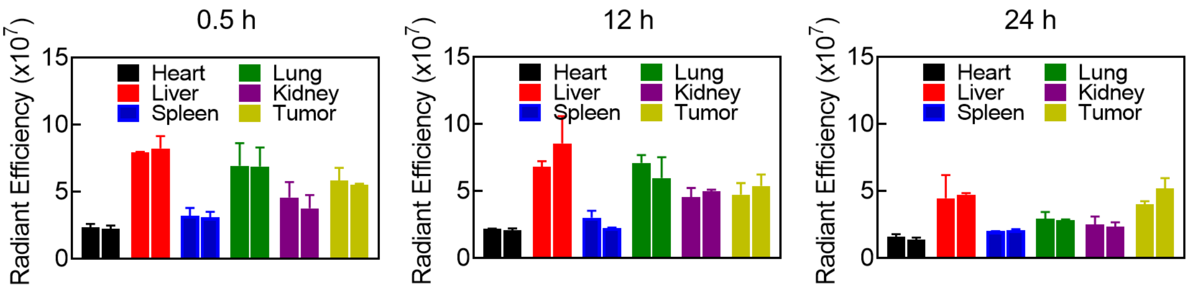


**Figure S6.** Quantitative results of fluorescence intensity in isolated tissues at different time point (n = 2). The numerical calculation results were presented as means ± SD.

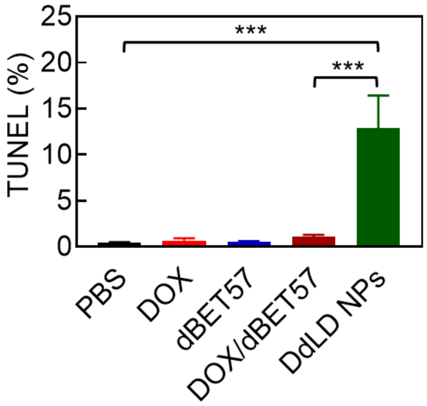


**Figure S7.** The TUNEL positive rate of tumor tissues after various treatments (n = 3). The numerical calculation results were presented as means ± SD. Statistical analysis was performed by one-way ANOVA. ****P* < 0.001.

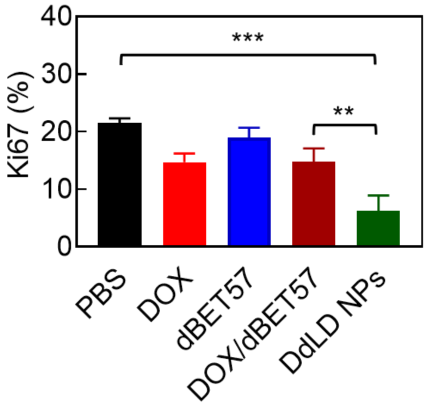


**Figure S8.** The Ki67 positive rate of tumor tissues after various treatments (n = 3). The numerical calculation results were presented as means ± SD. Statistical analysis was performed by one-way ANOVA. $^{**}P < 0.01$ and $^{***}P < 0.001$.

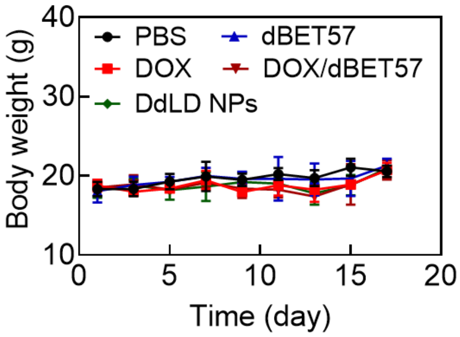


**Figure S9.** Body weights of CT26 tumor-bearing mice after various treatments (n = 5). The numerical calculation results were presented as means ± SD.

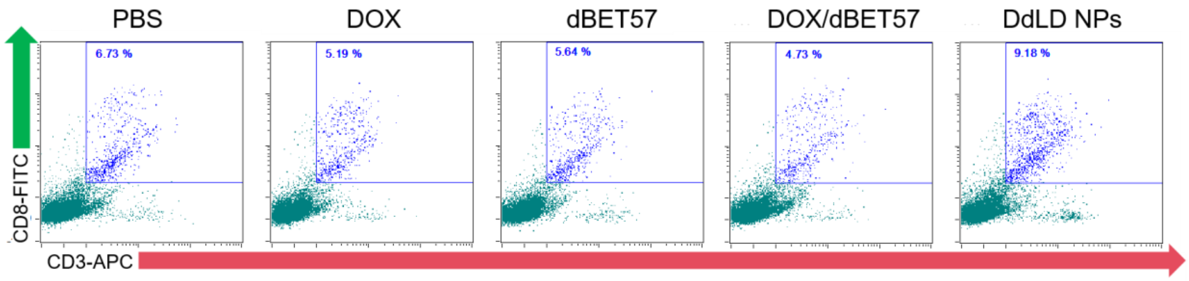


**Figure S10.** Representative images of flow cytometry analysis of $CD3^{+}$ $CD8^{+}$ T cells.

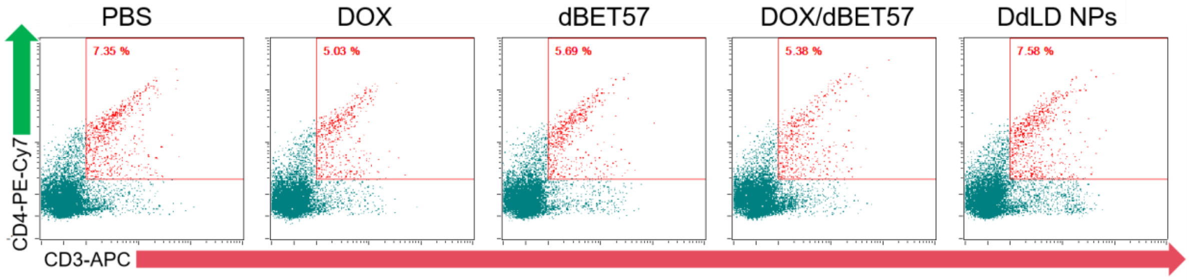


**Figure S11.** Representative images of flow cytometry analysis of $CD3^+$ $CD4^+$ T cells.

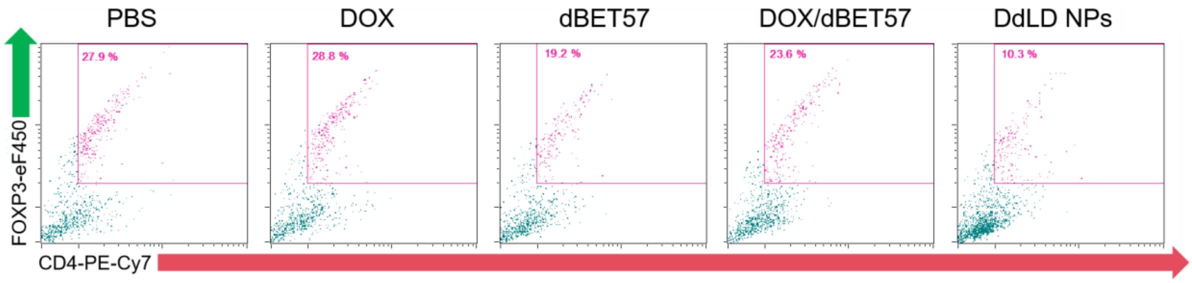


**Figure S12.** Representative images of flow cytometry analysis of treg cells.

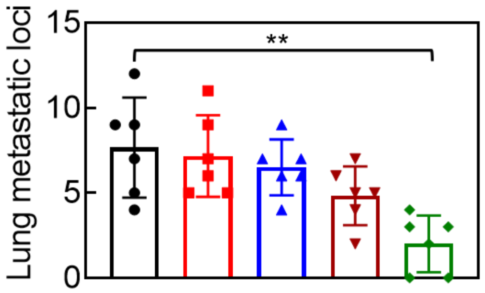


**Figure S13.** The number of lung metastatic loci in CT26 tumor-bearing mice (n = 6). The numerical calculation results were presented as means ± SD. Statistical analysis was performed by Student's t-test. $^{**}P < 0.01$.

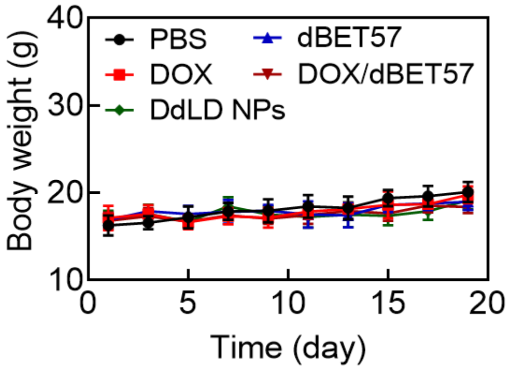


**Figure S14.** Body weights of CT26 tumor-bearing mice after various treatments (n = 6). The numerical calculation results were presented as means ± SD.

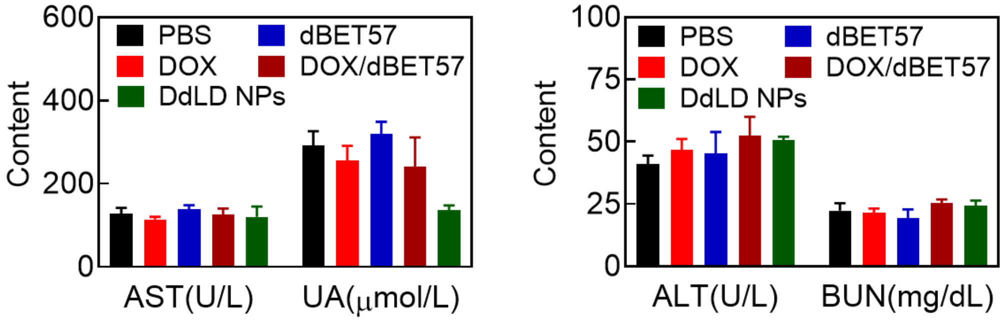


**Figure S15.** Blood biochemical indexs of CT26 tumor-bearing mice after various treatments (n = 3). The numerical calculation results were presented as means ± SD.
